# HIV-Associated Tuberculosis 2012

**DOI:** 10.1155/2012/950964

**Published:** 2012-10-04

**Authors:** K. A. Wilkinson, M. E. Torok, S. Schwander, G. Meintjes

**Affiliations:** ^1^National Institute for Medical Research, Mill Hill, London NW7 1AA, UK; ^2^Institute of Infectious Disease and Molecular Medicine, University of Cape Town, Rondebosch, Cape Town 7701, South Africa; ^3^Department of Infectious Diseases, Addenbrooke's Hospital, Cambridge CB2 0QQ, UK; ^4^Department of Medicine, Addenbrooke's Hospital, University of Cambridge, Box 157, Cambridge CB2 0QQ, UK; ^5^Department of Environmental and Occupational Health, Center for Global Public Health, School of Public Health, UMDNJ, NJ, USA

Tuberculosis (TB) is an important public health problem and remains a leading cause of death in low- and middle-income countries, with an estimated 9.27 million new cases and 1.7 million deaths worldwide in 2007. HIV infection is the greatest risk factor for developing TB. The devastating association between HIV and TB means that 1.37 million of the new TB cases were amongst HIV-infected people and one out of four TB deaths was HIV related in 2007. The risk of TB is increased during all stages of HIV infection from about 10% over a lifetime (in HIV-uninfected individuals) to as high as 30% per annum in patients with advanced HIV.

This special issue addresses several important aspects of the clinical presentation and management, diagnosis, and immunology of HIV-associated TB.

Combination antiretroviral therapy (cART) has progressively decreased mortality of HIV-associated tuberculosis patients worldwide. However, despite the increasing roll out of antiretroviral therapy (ART), the number of deaths attributable to TB still remains high in some parts of the world.

The paper by T. T. Liu et al. highlights this fact and the discrepancies between TB-attributed hospital deaths and the diagnostically confirmed TB deaths, emphasizing the difficulties of diagnosing TB in a high HIV prevalence setting in routine hospital care. The paper by E. Girardi et al. examined TB treatment outcomes in HIV-infected patients diagnosed with TB in Italy. ART during TB treatment was associated with substantial reduction of death rate, while patients who were not ART naïve when they developed TB had an elevated risk of death.

HIV-infected patients are more likely to develop extrapulmonary forms of TB, such as pleural TB. While pleural TB in the immune competent host may be a self-limiting disease, it provides formidable challenges to clinicians around the world in the context of HIV. The systematic review by A. Aljohaney et al. summarises the epidemiology, the immunopathogenesis, and the challenges associated with diagnosis and treatment of pleural TB in HIV-infected patients.

The high rate of latent TB infection is the main challenge to achieving global control of TB. The introduction of IGRA has greatly facilitated diagnosis of LTBI in the developed countries; however, the difficulty in developing countries and HIV-infected individuals remains. The antibody response to tubercular glycolipid (TBGL) antigen was shown to be a promising serodiagnostic agent in Japan, and the paper by U. R. Siddiqi et al. evaluates the antibody response against this antigen in health care workers and HIV-infected individuals in the Philippines.

Chronic HIV infection leads to excessive production of proinflammatory cytokines which leads to the generation of free radicals which are in turn scavenged by free glutathione. Thus the excessive production of free radicals in HIV-infected individuals or reduced glutathione synthesis may lead to the depletion of glutathione. D. Morris et al. examined the causes for decreased glutathione in individuals with chronic HIV infection and found lower levels of intracellular glutathione in macrophages and increased levels of free radicals, interleukin-1 (IL-1), interleukin-17 (IL-17), and transforming growth factor-*β* (TGF-*β*) in plasma samples. They also found reduced expression of enzymes responsible for glutathione synthesis. These findings may contribute to the loss of immune function observed in chronic HIV infection.

*K. A. Wilkinson* *M. E. Torok* *S. Schwander* *G. Meintjes*

